# Supplementary material for: In-context learning enables multimodal large language models to classify cancer pathology images
Source: Nat Commun. 2024 Nov 21;15:10104. doi: 10.1038/s41467-024-51465-9 (PMC11582649; doi:10.1038/s41467-024-51465-9)
Supplement: Supplementary file 3 — Reporting Summary [file 41467_2024_51465_MOESM3_ESM.pdf]

nature portfolio

Corresponding author(s): Dyke Ferber, Georg Wölflein, Isabella C. Wiest , Marta Ligero, Srividhya Sainath, Narmin Ghaffari Laleh, Omar S.M. El Nahhas, Gustav Müller-Franzes, Dirk Jäger, Daniel Truhn, Jakob Nikolas Kather

Last updated by author(s): July 24, 2024

Reporting Summary

Nature Portfolio wishes to improve the reproducibility of the work that we publish. This form provides structure for consistency and transparency in reporting. For further information on Nature Portfolio policies, see our Editorial Policies and the Editorial Policy Checklist.

Statistics

For all statistical analyses, confirm that the following items are present in the figure legend, table legend, main text, or Methods section.

n/a	Confirmed	
☐	☒	The exact sample size (*n*) for each experimental group/condition, given as a discrete number and unit of measurement
☒	☐	A statement on whether measurements were taken from distinct samples or whether the same sample was measured repeatedly
☐	☒	The statistical test(s) used AND whether they are one- or two-sided<br>*Only common tests should be described solely by name; describe more complex techniques in the Methods section.*
☒	☐	A description of all covariates tested
☐	☒	A description of any assumptions or corrections, such as tests of normality and adjustment for multiple comparisons
☐	☒	A full description of the statistical parameters including central tendency (e.g. means) or other basic estimates (e.g. regression coefficient) AND variation (e.g. standard deviation) or associated estimates of uncertainty (e.g. confidence intervals)
☐	☒	For null hypothesis testing, the test statistic (e.g. *F*, *t*, *r*) with confidence intervals, effect sizes, degrees of freedom and *P* value noted<br>*Give P values as exact values whenever suitable.*
☒	☐	For Bayesian analysis, information on the choice of priors and Markov chain Monte Carlo settings
☒	☐	For hierarchical and complex designs, identification of the appropriate level for tests and full reporting of outcomes
☒	☐	Estimates of effect sizes (e.g. Cohen's *d*, Pearson's *r*), indicating how they were calculated

Our web collection on statistics for biologists contains articles on many of the points above.

Software and code

Policy information about availability of computer code

Data collection	3 data sources were used in our study: Images for CRC100K and PatchCamelyon are publicly available on the internet; the MHIST dataset can be obtained for free upon request. No commercial or private (in-house) data was used in this study.
Data analysis	1. Proprietary models. We used OpenAI's GPT-4V model via their offical API. This requires an API key and a payment strategy as well as adhering to the official OpenAI license. For text-embeddings we used the proprietary ada-002 model from OpenAI.<br>2. Vision Models: Vision Models (ViT and Resnets) were trained using the open source PyTorch, timm and hugging-face library.<br>3. Embeddings: Embeddings for feature-vectors for kNN-sampling were generated in numpy.<br>4. Data analysis was performed with open-source community tools, including matplotlib, pandas, seaborn and plotly.

For manuscripts utilizing custom algorithms or software that are central to the research but not yet described in published literature, software must be made available to editors and reviewers. We strongly encourage code deposition in a community repository (e.g. GitHub). See the Nature Portfolio guidelines for submitting code & software for further information.

Data

Policy information about availability of data

All manuscripts must include a data availability statement. This statement should provide the following information, where applicable:

- Accession codes, unique identifiers, or web links for publicly available datasets
- A description of any restrictions on data availability
- For clinical datasets or third party data, please ensure that the statement adheres to our policy

The data that support our findings are publically available and can be downloaded from https://huggingface.co/datasets/DykeF/NCTCRCHE100K or https://zenodo.org/records/1214456 (NCT-CRC-HE-100K) and https://github.com/basveeling/pcam (PatchCamelyon). The MHIST dataset can be obtained for free upon request under https://bmirds.github.io/MHIST/ (MHIST).

Research involving human participants, their data, or biological material

Policy information about studies with human participants or human data. See also policy information about sex, gender (identity/presentation), and sexual orientation and race, ethnicity and racism.

Reporting on sex and gender	We did not perform sex- or gender based analyses in our study.
Reporting on race, ethnicity, or other socially relevant groupings	We did not examine racial, ethnical or other socially relevant information in our study.
Population characteristics	We used classical image benchmarking datasets for histopathology, without accounting for population characteristics.
Recruitment	n/a
Ethics oversight	The overall analysis was approved by the Ethics commission of the Medical Faculty of the Technical University Dresden (BO-EK-444102022).

Note that full information on the approval of the study protocol must also be provided in the manuscript.

Field-specific reporting

Please select the one below that is the best fit for your research. If you are not sure, read the appropriate sections before making your selection.

☒ Life sciences ☐ Behavioural & social sciences ☐ Ecological, evolutionary & environmental sciences

For a reference copy of the document with all sections, see nature.com/documents/nr-reporting-summary-flat.pdf

Life sciences study design

All studies must disclose on these points even when the disclosure is negative.

Sample size	We acknowledge to run our experiments on a small sample size, which is due to the preview-status of the GPT-4 Vision model that sets strict rate limits (number of requests per day). However we show a consistent improvement of the results by gradually increasing the number of few-shot samples we give to the model. Moreover we show that by bootstrapping over the results, we achieve no overlap in the Confidence Intervals (i.e. Table 1: GPT-4V on CRC100K with zero vs. three and zero vs. five few-shot samples).<br>For chosing the kNN- or random sampling sample-size we scaled up the number of image samples, that are shown to GPT-4 until we exhausted the maximum context window the model allows for. For instance, in the case of the NCT-CRC100K dataset, we were limited to using a maximum sample size of n=5 per label. Showing 10 images per label for 8 labels would mean we feed 81 images (8*10 + the image of interest) into the model prompt at a time, which led the request to be refused by OpenAI's API.
Data exclusions	In the MHIST dataset, we excluded images where there was no complete inter-rater agreement, meaning we only included images with a 0/7 or 7/7 vote by all seven pathologists.
Replication	Given the restricted API usage, we did not replicate our results but release all required source code in order to do so. For statistical analyses, we performed bootstrapping and report confidence intervals.
Randomization	To generate balanced splits, we assigned the same number of images for each label into the test datasets, for instance for NCT-CRC100K we had a total number of 120 samples (15 images for 8 labels). From the entire image dataset corpus, images were randomly drawn.
Blinding	Test datasets were created by randomly collecting images from the larger dataset cohort.

Reporting for specific materials, systems and methods

We require information from authors about some types of materials, experimental systems and methods used in many studies. Here, indicate whether each material, system or method listed is relevant to your study. If you are not sure if a list item applies to your research, read the appropriate section before selecting a response.

Materials & experimental systems

n/a	Involved in the study
☒	☐ Antibodies
☒	☐ Eukaryotic cell lines
☒	☐ Palaeontology and archaeology
☒	☐ Animals and other organisms
☒	☐ Clinical data
☒	☐ Dual use research of concern
☒	☐ Plants

Methods

n/a	Involved in the study
☒	☐ ChIP-seq
☒	☐ Flow cytometry
☒	☐ MRI-based neuroimaging

Plants

Seed stocks	*Report on the source of all seed stocks or other plant material used. If applicable, state the seed stock centre and catalogue number. If plant specimens were collected from the field, describe the collection location, date and sampling procedures.*
Novel plant genotypes	*Describe the methods by which all novel plant genotypes were produced. This includes those generated by transgenic approaches, gene editing, chemical/radiation-based mutagenesis and hybridization. For transgenic lines, describe the transformation method, the number of independent lines analyzed and the generation upon which experiments were performed. For gene-edited lines, describe the editor used, the endogenous sequence targeted for editing, the targeting guide RNA sequence (if applicable) and how the editor was applied.*
Authentication	*Describe any authentication procedures for each seed stock used or novel genotype generated. Describe any experiments used to assess the effect of a mutation and, where applicable, how potential secondary effects (e.g. second site T-DNA insertions, mosiacism, off-target gene editing) were examined.*
